# Clinical characteristics that distinguish eosinophilic organ infiltration from metastatic nodule development in cancer patients with eosinophilia

**DOI:** 10.1186/1477-7819-10-175

**Published:** 2012-08-28

**Authors:** Taehoon Lee, Yoon Su Lee, Sun Young Yoon, Su-Jeong Kim, Yun-Jeong Bae, Hyouk-Soo Kwon, You Sook Cho, Hee-Bom Moon, Tae-Bum Kim

**Affiliations:** 1 Division of Allergy and Clinical Immunology, Department of Internal Medicine, Asan Medical Center, University of Ulsan College of Medicine, 388-1 Pungnap-2dong Songpa-gu, Seoul, 138-736, South Korea

**Keywords:** Eosinophilia, Liver abscess, Pulmonary eosinophilia, Neoplasms

## Abstract

**Background:**

When new space-occupying lesions are observed together with peripheral blood eosinophilia in patients diagnosed with cancer, the possibility of eosinophilic organ involvement should be differentiated from metastasis of primary cancer, since a misdiagnosis could lead to unnecessary chemotherapy. The aim of this study is to identify the clinical characteristics of eosinophilic organ involvement that distinguish it from distant metastasis in patients with primary cancer.

**Methods:**

The medical records of 43 cancer patients who developed hepatic or pulmonary nodules with peripheral blood eosinophilia between January 2005 and February 2010 in the Asan Medical Center (Seoul) were reviewed. Eosinophilic infiltration and distant metastasis were identified on the basis of pathological findings and radiological features. Fisher’s exact test, *χ*^2^ test or Mann-Whitney test were used for statistical analysis.

**Results:**

In total, 33 patients (76%) were diagnosed with eosinophilic infiltration, 5 (12%) with cancer metastasis and 5 (12%) had undetermined diagnoses. Compared to the patients with metastases, the patients with eosinophilic infiltration were significantly more likely to have serology indicating a parasitic infection, a history of eating raw food, high serum levels of total IgE, normal liver function, normal C-reactive protein levels, a normal erythrocyte sedimentation rate, and fewer and smaller nodules. The most common underlying malignancy in the eosinophilic organ infiltration group was stomach cancer. Physicians tended to neglect the eosinophilia in patients with a history of cancer.

**Conclusions:**

Several clinical characteristics of eosinophilic organ infiltration distinguish it from cancer metastasis. Physicians should make greater efforts to determine the causes of organ involvement with peripheral blood eosinophilia, especially in cancer patients.

## Background

Eosinophilic organ infiltration often damages target organs and induces their dysfunction by releasing cytotoxic granule proteins and inflammatory lipid mediators and promoting thromboembolic phenomena [1,2]. Even though eosinophilic expansion is secondary to an identifiable disease (such as parasitic infection, drug hypersensitivity, allergic diseases, collagen vascular diseases and internal malignancies), the damage it can cause by organ infiltration can affect various organ systems, including the skin, lung, liver, brain and, most importantly, the heart [3-6]. However, most cases of eosinophilic organ infiltration can be cured easily by correcting the underlying cause of the eosinophilia [7].

One particularly challenging problem in clinical practice is when a patient with cancer develops eosinophilic organ infiltration. Since the features of eosinophilic organ infiltration can mimic those of cancer metastasis, it can lead to a misdiagnosis and unnecessary anti-cancer therapy. Indeed, misdiagnoses of eosinophilic abscesses as metastases have been described in anecdotal case reports [8-12].

Several reports describe how to identify eosinophilic organ infiltration by radiology [13-20]. However, the clinical characteristics of eosinophilic organ infiltration in patients with a history of cancer have not been researched previously. This is important because this information could aid the management of patients with a history of cancer who present with a newly developed nodule and peripheral eosinophilia.

In this study, patients with a history of cancer who developed new nodules in the liver or lung and exhibited peripheral eosinophilia were identified retrospectively. The clinical characteristics of eosinophilic organ involvement that distinguished it from metastasis were then determined. In addition, the diagnostic trials that were performed by the physicians to determine the cause of the eosinophilia and organ infiltration were analyzed.

## Methods

### Study subjects

**Figure 1:**
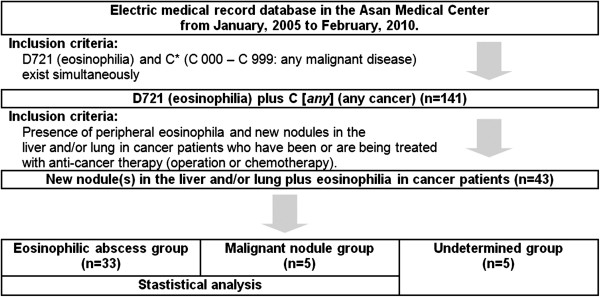
Selection of the cases for the final analysis.

The medical records of patients who visited or were admitted to the Asan Medical Center (Seoul) between 1 January 2005 and 28 February 2010 were assessed using the International Statistical Classification of Diseases and Related Health Problems, 10th Revision (ICD-10). The patients whose ICD-10 codes included both D 721 (eosinophilia) and C*(C 000 – C 999: any malignant disease) were selected (n = 141) (Figure 1). The records of these patients were then meticulously reviewed for the presence of peripheral eosinophilia and new nodules in the liver or lung of cancer patients who had been or were being treated with anti-cancer therapy (operation or chemotherapy). Of the 141 patients, 98 were excluded because of the absence of new nodules. The remaining 43 patients with a history of malignancy had both peripheral eosinophilia and newly developed nodules in the liver or lung (Figure 1).

### Study design

Demographic and clinical data, namely age, sex, duration of cancer, type of cancer, a history of eating raw foods, radiological data [the organ involved (liver and/or lung), the number of nodules in the involved organs and the diameter of the largest nodule] and laboratory data [liver function test results, eosinophil count, total IgE level, C-reactive protein (CRP) level, erythrocyte sedimentation rate (ESR), presence of parasite ova in the stools and parasite serology], were collected. Parasite serology was performed for *Paragonimus westermani, Clonorchis sinensis*, sparganosis, cysticercosis and toxocariasis by using enzyme-linked immunosorbent assays (ELISA).

Based on the pathological, radiological and clinical features of the new nodules in the liver or lung, the patients were grouped into the eosinophilic abscess group, the malignant nodule group or the undetermined diagnosis group (Figure 1). An eosinophilic abscess was defined as a biopsy-proven (including operative resection) eosinophilic abscess or a clinically eosinophilic abscess. A clinically eosinophilic abscess was diagnosed when all of the following conditions were satisfied: (1) the biopsy was not diagnostic or was not performed, and (2) serial enhanced computed tomography (CT) or ultrasonography imaging studies revealed the disappearance or spontaneous regression of the initially identified poorly circumscribed low-attenuation lesion (on CT) or low-echoic lesion (on US) that had been diagnosed by the radiologist as an eosinophilic abscess. Malignant nodules were defined as biopsy-proven (including operative resection) malignancies or as clinically malignant nodules. A clinically malignant nodule was diagnosed when all of the following conditions were satisfied: (1) the biopsy was not diagnostic or was not performed, and (2) serial CT or US studies revealed the progression of the initially identified well-circumscribed low-attenuation lesion (on CT) or low-echoic lesion (on US) that had been diagnosed by the radiologist as a malignancy (or, in the case of patients who expired before follow-up imaging studies could be performed, a single imaging study that led the radiologist to diagnose the lesion as a malignancy). The nodule was classified as “undetermined” if any of these criteria were not met.

The eosinophilic abscess and malignant nodule groups were also subdivided according to the probable cause of eosinophilia, namely (1) a parasite, (2) a drug (such as antibiotics or chemotherapeutic agents) and (3) unknown (this included cancer-induced eosinophilia). A parasitic etiology was selected as the cause when the following two conditions were satisfied: (1) the parasite serology returned a positive result, ova were observed in the stools, or there were high total IgE levels plus a history of eating raw foods, and (2) there was no other possible cause such as drugs. A drug etiology was selected when the eosinophilia developed within 4 weeks of drug exposure and there was no historical or laboratory evidence of a parasite infection. An unknown etiology was selected if (1) the cause was not known despite sufficient history-taking and laboratory testing or (2) the history-taking and laboratory testing were not sufficient for elucidating the cause of eosinophilia.

Finally, the diagnostic trials that were performed by the physicians to identify the causes of the eosinophilia and organ nodules were assessed. The trials were deemed ‘adequate’ if the physicians had asked about the history of eating raw foods and had ordered blood tests such as parasite ELISAs. The trials were deemed ‘inadequate’ if the physician had not asked about the history of eating raw foods and had not ordered blood tests such as parasite ELISAs.

### Statistical methods

Fisher’s exact test and *χ*^2^ test were performed for categorical variables, and the Mann-Whitney test was carried out for continuous variables. *P* < 0.05 was considered to indicate statistical significance.

## Results and discussion

Of the 43 patients with a history of cancer who displayed a new nodule(s) in the liver or lung along with eosinophilia, 33 were diagnosed with an eosinophilic abscess (5, biopsy-proven; 28, clinically) and 5 with a malignant nodule (2, biopsy proven; 3, clinically). The diagnosis of the remaining five patients could not be determined, and their data were excluded from further analysis. The eosinophilic abscess and malignant nodule groups did not differ significantly in terms of age, sex, duration after the cancer diagnosis and the organ involved (Tables 1 and 2). However, the patients with an eosinophilic abscess were more likely to have a history of eating raw food than the patients with malignant nodules (36% *vs.* 0%, *p* = 0.048). In addition, the eosinophilic abscess group had significantly fewer nodules than the malignant nodule group (3.9 ± 3.6 *vs.* 14.2 ± 8.6, *p* = 0.017). The nodules in the eosinophilic abscess group were also significantly smaller than those in the malignant nodule group (1.6 ± 0.6 *vs.* 6.2 ± 3.4 cm, *p* = 0.013). As expected, given the definitions of the two groups (see Materials and Methods), the nodules of the eosinophilic group either disappeared or decreased upon a follow-up imaging study, while the nodules of the malignant nodule group grew (Table 2). In one of the patients in the malignant nodule group, the eosinophilia and nodule disappeared at the same time, namely, the eosinophilia resolved when the nodule was resected.

**Table 1:** Demographic data of cases with eosinophilic abscesses and malignant nodules

**Characteristics**	**Eosinophilic abscess group (*n*** **= 33)**	**Malignant nodule group (*n*** **= 5)**	**P-value**
Age, years	54.6 ± 9.9	61.2 ± 16.2	0.243
Sex			1.000
Male	78 (26/33)	80 (4/5)	
Female	22 (7/33)	20 (1/5)	
Duration since diagnosis of cancer, years	2.4 ± 2.3	0.8 ± 1.3	0.182
History of eating raw food			0.048
Present	36 (12/33)	0 (0/5)	
Absent	6 (2/33)	40 (2/5)	
Unknown	58 (19/33)	60 (3/5)	

The data are presented as means ± SD or percentages (number of patients).

The *P*-values were determined by the Mann–Whitney *U*-test, chi-square or Fisher’s test.

**Table 2:** Radiological data of cases with eosinophilic abscesses and malignant nodules

**Characteristics**	**Eosinophilic abscess group (*n*** **= 33)**	**Malignant nodule group (*n*** **= 5)**	**P-value**
Organ that was involved			0.599
Liver only	85 (28/33)	80 (4/5)	
Lung only	6 (2/33)	20 (1/5)	
Liver and lung	9 (3/33)	0 (0/5)	
Nodules in the organs involved, number	3.9 ± 3.6	14.2 ± 8.6	0.017
Diameter of the largest nodule, cm	1.6 ± 0.6	6.2 ± 3.4	0.013
Duration of the nodules, months	10.0 ± 8.9		
Status of the nodule(s) in a follow-up study,			< 0.001
Totally disappeared	91 (30/33)	20 (1/5)*	
Decreased	9 (3/33)	0 (0/5)	
Increased	0 (0/33)	80 (4/5)	

*Operative resection was performed in the case where the malignant nodule disappeared.

The data are presented as means ± SD or percentages (number of patients).

The *P*-values were determined by the Mann-Whitney *U*-test, chi-square or Fisher’s test.

The eosinophilic abscess group had significantly higher serum total IgE levels than the malignant nodule group (7.4 ± 1.4 *vs.* 4.9 ± 1.2 log IgE, *p* = 0.021) (Table 3). The eosinophilic abscess group also had significantly lower liver function test results [AST (aspartate transaminase), ALT (alanine transaminase), alkaline phosphatase and total bilirubin] and CRP and ESR levels than the malignant nodule group: AST (26.6 ± 12.9 IU/l *vs.* 112.60 ± 100.0 IU/l, *p* = 0.002), ALT (22.7 ± 18.7 IU/l *vs.* 80.0 ± 47.4 IU/l, p = 0.002), alkaline phosphatase (76.4 ± 23.1 IU/l *vs.* 460.0 ± 509.8 IU/l, *p* = 0.019), total bilirubin (0.9 ± 0.4 mg/dl *vs.* 5.2 ± 8.3 mg/dl, *p* = 0.002), CRP (0.8 ± 0.9 mg/dl *vs.* 10.8 ± 12.8, *p* = 0.004), and ESR (31.3 ± 19.1 mm/h *vs.* 102.5 ± 24.8 mm/h, *p* = 0.040) (Table 3).

**Table 3:** Laboratory data of cases of eosinophilic abscesses and malignant nodules

**Characteristics**	**Eosinophilic abscess group (*n*** **= 33)**	**Malignant nodule group (*n*** **= 5)**	**P-value**
Blood eosinophils, number/ul	1,808.6 ± 1,625.8	4,186 ± 4,053.7	0.160
Blood eosinophils, %	20.0 ± 11.2	31.6 ± 21.0	0.243
Severity of eosinophilia,			0.060
Mild (450–1,500/ul)	52 (17/33)	40 (2/5)	
Moderate (1,500–5,000/ul)	45 (15/33)	20 (1/5)	
Severe (5,000–/ul)	3 (1/33)	40 (2/5)	
Duration of eosinophilia, months	10.5 ± 9.8		
Log total IgE	7.4 ± 1.4	4.9 ± 1.2	0.021
AST, IU/l	26.6 ± 12.9	112.60 ± 100.0	0.002
ALT, IU/l	22.7 ± 18.7	80.0 ± 47.4	0.002
Alkaline phosphatase, IU/l	76.4 ± 23.1	460.0 ± 509.8	0.019
Bilirubin, total, mg/dl	0.9 ± 0.4	5.2 ± 8.3	0.002
C-reactive protein, mg/dl	0.8 ± 0.9	10.8 ± 12.8	0.004
Erythrocyte sedimentation rate, mm/h	31.3 ± 19.1	102.5 ± 24.8	0.040

The data are presented as means ± SD or number (%).

The *P*-values are determined by the Mann-Whitney *U*-test, chi-square or Fisher’s test.

**Figure 2:**
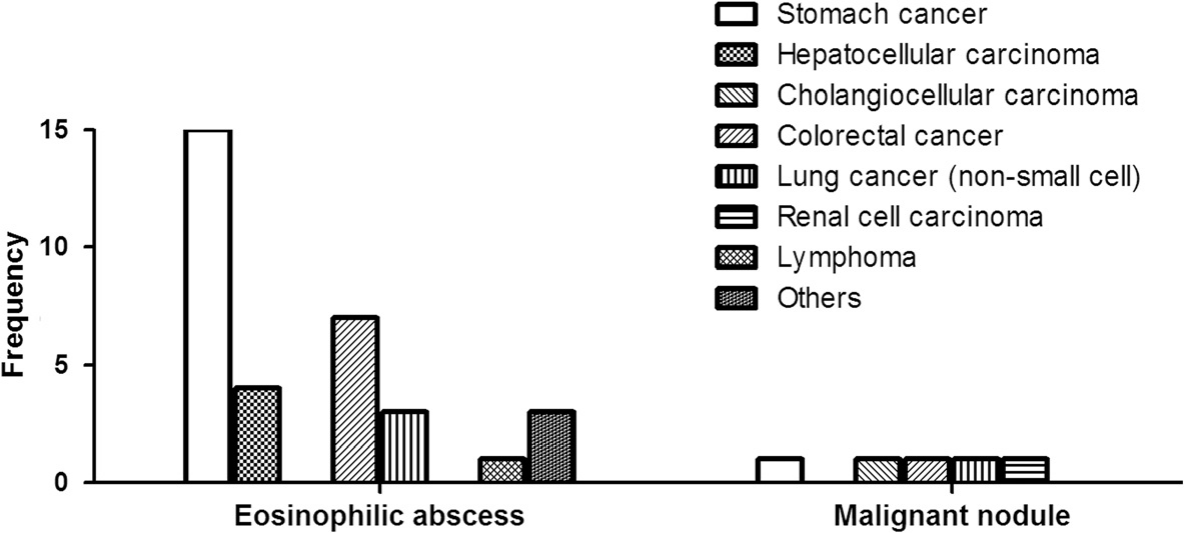
The types of underlying malignancy in the eosinophilic abscess and malignant nodule groups.

Stomach cancer was the most common malignant disease that was associated with eosinophilic abscess [46% (15/33)]. The other underlying malignancies in the patients with eosinophilic abscess were, in order of frequency, colorectal cancer, hepatocellular carcinoma and lung cancer (Figure 2).

**Figure 3:**
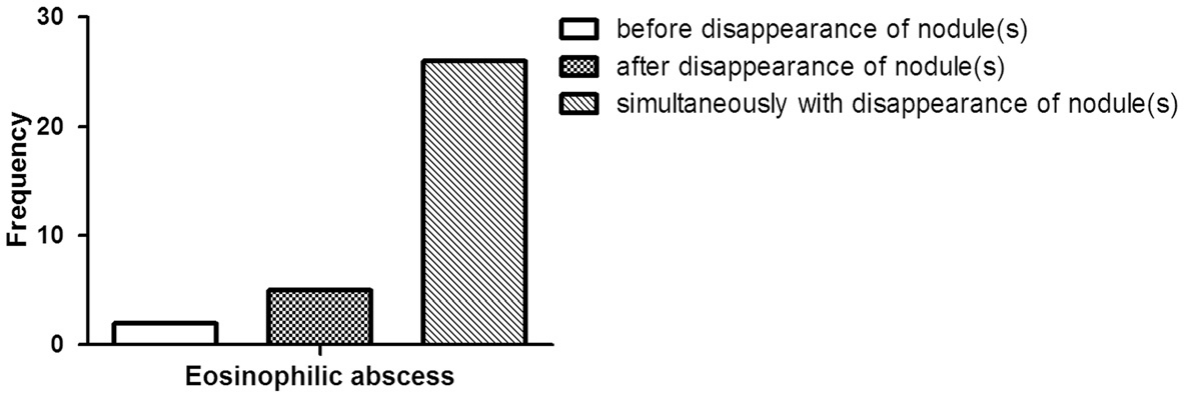
Resolution of eosinophilia in the eosinophilic abscess.

The diagnosis of an eosinophilic abscess required that it eventually regressed. An analysis of the relationship between the disappearance of both the nodule and the eosinophilia in eosinophilic abscess patients revealed that the peripheral eosinophilia was more likely to normalize at the same time that the eosinophilic abscess disappeared rather than before or after (Figure 3).

**Figure 4:**
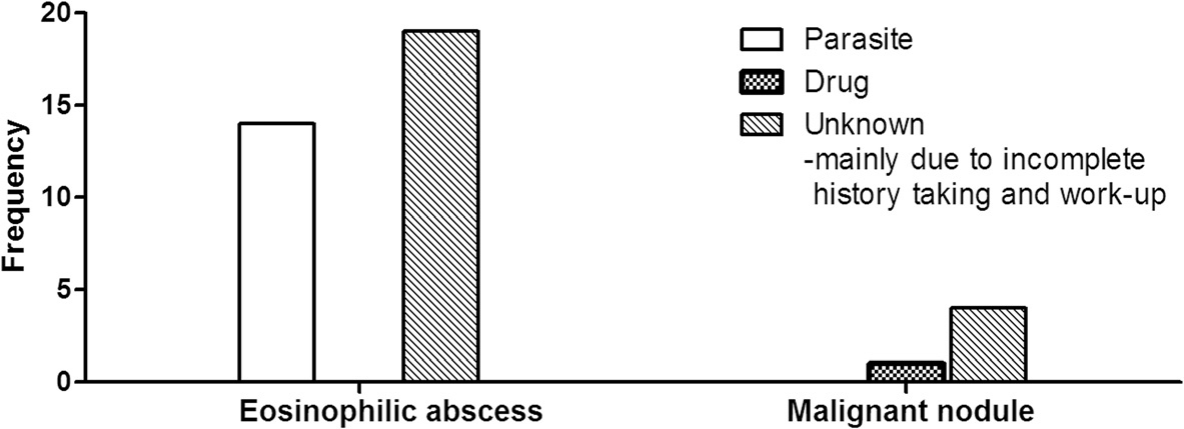
Presumptive etiology of the eosinophilia in the eosinophilic abscess and malignant nodule groups.

The cause of eosinophilia was most often unknown in both groups, mainly because of incomplete history taking and workup (Figure 4): in the eosinophilic abscess group, the causes were either undetermined (*n* = 19) or due to a parasite (*n* = 14) (none of the patients had a drug-related cause); in the malignant nodule group, the causes were undetermined in four patients and due to a drug in the remaining patient. Thus, a parasite was significantly more frequently the cause of eosinophilia in the eosinophilic abscess group than in the malignant nodule group [42% (14/33) *vs.* 0% (0/5), *p* = 0.04] (Figure 4).

**Figure 5:**
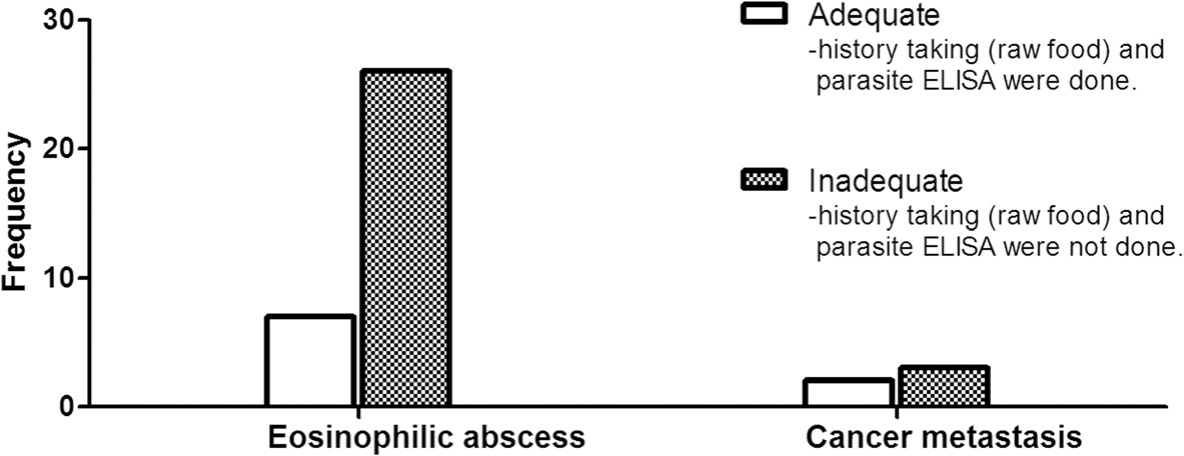
The degree of effort to find out the cause of eosinophilia in the eosinophilic abscess and malignant nodule groups.

The degree of effort made by the physicians to determine the cause of eosinophilia was most frequently ‘inadequate’ in both groups [eosinophilic abscess group, 79% (26/33); malignant nodule group, 60% (3/5), *p* = 0.574] (Figure 5).

The present study showed that eosinophilic organ infiltration can be distinguished from metastasis in cancer patients with eosinophilia on the basis of several clinical characteristics. First, parasite infestation was a significant cause of eosinophilic organ infiltration. Many patients with eosinophilic organ infiltration had a history of eating raw meat and had high total IgE levels and positive parasite ELISA results, unlike the malignant nodule group patients. In Korea, there is a custom of ingesting raw cow liver, which is a reservoir for the encapsulated larvae of *Toxocara cani*; consequently, ingestion of raw cow liver induces human toxocariasis [14,15]. The direct parasite invasion in toxocariasis results in the ensuing eosinophilia. *Toxocara cani*, along with other parasites such as trematodes, also generate focal lesions in the hepatic parenchyma, either by directly penetrating the liver or *via* hematogenous migration to the liver [20]. These lesions are caused by arrested immature worms and thus contain worms. Parasite-induced secondary eosinophilia more frequently involves the liver or lung than the heart, skin or nervous system and seems to induce nodules in those organs [8-13,15-20,22,23].

The second clinical feature that distinguishes eosinophilic organ infiltration from malignant nodules is one that was used to diagnose the patients in this study, namely, the different radiological findings. Thus, on CT or US, the eosinophilic infiltrations and the malignant nodules are both depicted as low-attenuation or low-echoic lesions, respectively. However, the eosinophilic abscesses are poorly demarcated, multifocal, and small (< 2 cm) and either disappear or regress considerably upon subsequent CT or US imaging studies. In contrast, the malignant nodules are well circumscribed, significantly larger and generally progress. These findings are consistent with those of previous studies [18,20]. In addition, the present study found that while the hepatic eosinophilic infiltrations were multifocal, there were far fewer nodules than in malignant nodule group patients. The mean duration of eosinophilic infiltration was about 10 months, and the nodules tended to disappear at the same time that the eosinophilia normalized.

The third clinical feature that distinguishes eosinophilic infiltrations was that the liver functions and CRP and ESR levels were significantly lower (namely within normal limits) in the eosinophilic infiltration patients than in the patients with malignant nodules. It seems that the eosinophils destroy the hepatic parenchyma less profoundly than malignant cells.

Fourth, the most common underlying malignancy in patients with eosinophilic organ infiltration was stomach cancer. This was also observed in another study [24]. Although the reason for this is not clear, some tumors are known to produce eosinophilotactic factors [21]. It may be that stomach cancer cells produce particularly large amounts of such factors.

Lastly, we observed that the physicians tended to neglect the eosinophilia in patients with history of a cancer, as the degree of effort made by the physicians to determine the cause of the eosinophilia was largely inadequate. This could result in the misdiagnosis of an eosinophilic abscess as a malignant nodule, which in turn could lead to unnecessary chemotherapy. In fact, we have observed the case of a patient who had an eosinophilic abscess that was indeed misdiagnosed as a relapsed malignancy (unpublished observation). Given that eosinophilic organ infiltration can be cured easily by correcting the underlying etiology of the eosinophilia without unnecessary chemotherapy [7], our observation suggests that physicians should make a greater effort to determine the cause of eosinophilia, especially in cancer patients who have both new nodules and peripheral eosinophilia.

The present study had some limitations. First, the eosinophilic abscess was not pathologically proven in all patients. However, in cases where there was no histological proof, the disease was diagnosed on the basis of the characteristic imaging features of eosinophilic abscesses and follow-up imaging studies that showed that the lesions regressed partially or completely. Second, the study had a retrospective design. Therefore, there were many missing values, and this made the statistical analysis difficult. Third, since the malignant nodule group (*n* = 5) was too small for proper comparison with the eosinophilic abscess group (*n* = 33), the results should be interpreted cautiously. Another large-sized study is required to confirm these results.

## Conclusion

This study is the first to report the clinical characteristics that may distinguish eosinophilic organ involvement from metastasis in cancer patients with eosinophilia. It also shows that physicians should make greater efforts to determine the cause of eosinophilia in such patients, especially those who have both new nodules and peripheral eosinophilia. This would obviate the chance of unnecessary chemotherapy. Moreover, most cases of eosinophilic organ infiltration can be cured easily by correcting the etiology underlying the eosinophilia.

## Consent

This retrospective study was reviewed and approved by the institutional review boards of Asan Medical Center, University of Ulsan.

## Competing interests

The authors declare that they have no competing interests.

## Authors’ contributions

TL conducted the clinical assessments, analyzed and interpreted data and wrote the first draft; YSL, SYY, SJK, YJB and HSK contributed to data collection and interpretation; YSC and HBM advised on and helped with the study design, contributed to data analysis and interpretation; TBK conceived the study and design, advised on and helped with study design, discussed core ideas, designed data collection protocols, and helped with data interpretation and writing. All authors read and approved the final manuscript.
